# Utilizing Iron for Targeted Lipid Peroxidation as Anticancer Option of Integrative Biomedicine: A Short Review of Nanosystems Containing Iron

**DOI:** 10.3390/antiox9030191

**Published:** 2020-02-25

**Authors:** Morana Jaganjac, Suzana Borovic Sunjic, Neven Zarkovic

**Affiliations:** 1 Qatar Analytics & BioResearch Laboratory, Anti Doping Laboratory Qatar, Doha, Qatar; mjaganjac@adlqatar.qa; 2 Rudjer Boskovic Institute, Laboratory for Oxidative Stress, Division of Molecular Medicine, Bijenicka 54, 10000 Zagreb, Croatia; borovic@irb.hr

**Keywords:** iron, nanomedicine, reactive oxygen species, ferroptosis, 4-hydroxynonenal

## Abstract

Traditional concepts of life sciences consider oxidative stress as a fundamental process of aging and various diseases including cancer, whereas traditional medicine recommends dietary intake of iron to support physiological functions of the organism. However, due to its strong pro-oxidative capacity, if not controlled well, iron can trigger harmful oxidative stress manifested eventually by toxic chain reactions of lipid peroxidation. Such effects of iron are considered to be major disadvantages of uncontrolled iron usage, although ferroptosis seems to be an important defense mechanism attenuating cancer development. Therefore, a variety of iron-containing nanoparticles were developed for experimental radio-, chemo-, and photodynamic as well as magnetic dynamic nanosystems that alter redox homeostasis in cancer cells. Moreover, studies carried over recent decades have revealed that even the end products of lipid peroxidation, represented by 4-hydroxynonenal (4-HNE), could have desirable effects even acting as kinds of selective anticancer substances produced by non-malignant cells for defense again invading cancer. Therefore, advanced nanotechnologies should be developed for using iron to trigger targeted lipid peroxidation as an anticancer option of integrative biomedicine.

## 1. Introduction

One of the most challenging health conditions is cancer, and nanotechnology has provided new opportunities for the development of more efficient treatment procedures. The potential biomedical benefits of nanoparticles were first suggested in the 1970s, and the era of nanomedicine started at the turn of this century. Iron containing nanoparticles including iron oxide nanoparticles (IONPs), are promising tools for anticancer therapies. On the basis of the different oxidation states and crystalline structures, IONPs comprise magnetite (Fe_3_O_4_), maghemite (γ-Fe_2_O_3_), and hematite (α-Fe_2_O_3_). IONPs made up of magnetite and maghemite have great biocompatibility with superparamagnetic properties. Recently, a variety of iron containing nanosystems, as potent agents in anticancer treatment, have been described in the literature. Iron oxide nanoparticle properties have been reported to be useful for various biomedical purposes, such are targeted drug delivery, bioimaging, biosensors, and hyperthermia [1], while some are approved by Food and Drug Administration (FDA) [2,3]. Hence, NanoTherm for management of glioblastoma and Ferumoxytol for iron deficiency treatment are among the FDA approved iron oxide containing nanomedicines.

Iron has crucial roles in biological systems, such as the immune system, neural system, and muscular system. Iron also plays an important role in carcinogenesis and altered iron metabolism in cancer has been well documented [4,5]. Some of the regulatory roles of iron are described below.

## 2. Iron Regulated Cellular Processes

**Figure 1:**
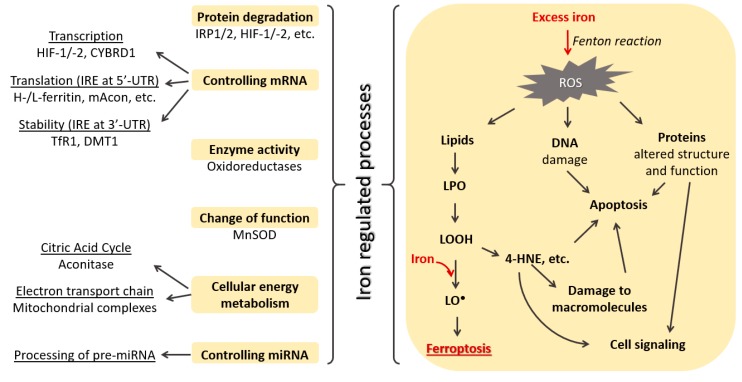
Some cellular processes regulated by iron.

Iron is an essential nutrient and in the form of ferric ions (Fe^3+^) is bound to transferrin and carried through the body. When diferric transferrin is bound to the transferrin receptor the complex is endocytosed. In the endosome, Fe^3+^ is reduced to ferrous iron (Fe^2+^) by a metalloreductase six-transmembrane epithelial antigen of prostate 3 (STEAP3). This is followed by Fe^2+^ translocation to cytoplasm via divalent metal transporter 1 (DMT1), where it enters a transient pool of metabolically active iron [6]. Intracellularly iron can regulate a variety of processes (Figure 1).

Iron can modulate cellular energy metabolism by influencing the citric acid cycle and oxidative phosphorylation [7]. Iron promotes the activity of citric acid cycle enzymes, such are mitochondrial aconitase (mAcon), citrate synthase, isocitric dehydrogenase, and succinate dehydrogenase [7]. When iron is transported to the mitochondria it can be utilized for heme synthesis or iron–sulfur (Fe-S) cluster formation which are crucial components of mitochondrial complexes and other proteins needed for mitochondrial respiration and ATP generation [8,9,10].

Another important role of iron is its ability to control mRNA transcription, translation, and stability [6]. Low iron concentration promotes hypoxia-inducible factor (HIF) transcription, while under conditions of iron repletion they are hydroxylated by the action of HIF prolyl hydroxylase domain proteins (PHDs) followed by protein degradation [11]. Furthermore, changes in iron concentration regulate mRNA containing an iron responsive element (IRE) in the 3′ and 5′ untranslated regions (UTRs). Binding of iron-regulatory protein (IRP) to IRE located in the 3′-UTR of the mRNA for transferrin receptor 1 [12] and DMT1 [13] enhances its stability against nuclease attack. IRPs also have high affinity towards IRE located at the 5′-UTR of the mRNA for mitochondrial aconitase [14] and heavy and light ferritin [15], suppressing its translation. These processes are described in detail in an excellent review by Lawen and Lane [6]. Additionally, iron is also involved in the control of processing of miRNA precursors through poly(C)-binding protein 2, affecting miRNA pathway activity [16]. 

In addition to being an essential nutrient, iron can also have potential toxic effects on cells. The toxicity is due to their pro-oxidant capacities leading to the generation of reactive oxygen species (ROS) that can affect macromolecules and alter cell signaling pathways [17]. It has been well recognized that ROS act as a double edge sword and that their activity depends on the concentration and type of ROS, as well as on their diffusion distance from the target molecules [18,19]. Excessive ROS, based on the amount, have dual effects on tumor growth [20]. Exposure of cervical cancer cells to Fe^3+^ induces ROS formation at lower concentrations and affects oncogene E6/E7 expression [21]. Iron and the consequent ROS were shown to modulate nuclear factor-kappa B (NF-κB) signaling pathway [22]. By inducing NF-κB signaling and affecting serum interleukin-6 and serotonin levels, iron exhibits an important immunomodulatory role [23].

Recently, it was reported that, under both physiological and pathological conditions, iron can be incorporated into mitochondrial superoxide dismutase (SOD)2. Iron incorporation in SOD2 leads to a switch in the enzyme activity from antioxidant function of superoxide dismutase to prooxidant peroxidase. This switch can further contribute to iron toxicity by affecting cellular signaling and metabolism and eventually the development of cancer [24].

Under normal physiological conditions, superoxide (O2●^−^) and hydrogen peroxide (H_2_O_2_), derived from a variety of cellular processes, are detoxified by cellular antioxidant systems. The O2●^−^ is first converted to H_2_O_2_, either spontaneously or by the action of SOD, while glutathione peroxidase (GPX), thioredoxin peroxidase, and catalase further decompose H_2_O_2_ to molecular oxygen and water. However, in an excess of iron, O2●^−^ and H_2_O_2_ take part in the Fenton reaction yielding highly reactive hydroxyl radical (OH●) [25]. Additionally, iron promotes the generation of reactive nitrogen species (RNS). Both, RNS and OH● promote damage of proteins and lipid peroxidation [26,27,28,29]. Depending on the amount of lipid peroxidation products formed, they have either an important signaling role or exhibit cytotoxic effects for the cells and tissues [30,31]. The consequences of iron induced lipid peroxidation are described in detail below. Thus, it is evident that maintaining iron and redox homeostasis is essential. 

## 3. Ferroptosis as a Target for Oncotherapy

Some of the mechanisms of the action of iron containing anticancer therapies account for their ability to induce ferroptosis (Figure 1). In 2012, ferroptosis was defined as a regulated nonapoptotic and iron dependent form of cell death [32], which depends on the intracellular concentration of iron as well as on the amount of ROS and level of lipid peroxidation. The process of ferroptosis is induced by the inhibition of cysteine import, inactivation of the phospholipid peroxidase glutathione peroxidase 4 (GPX4) activity and glutathione (GSH) depletion, lipid peroxidation, and excessive accumulation of lipid-based ROS (LO●) [33,34]. Today, different classes of ferroptosis induction compounds (FINs) are recognized [35]. Those responsible for inducing ferroptosis by depletion of intracellular glutathione are classified as Class I FINs. Ferroptosis can be triggered by GPX4 inactivation either directly (Class II FINs) or indirectly (Class III FINs). Additionally, an increase in the LIP via increased activity of heme oxygenase or directly via iron overload, can also trigger ferroptosis (Class IV FINs) [35].

An abundance of cellular NADPH can determine the sensitivity to ferroptosis as it has an important role in the elimination of lipid hydroperoxides (LOOH). Nevertheless, the abundance of NADP(H) has been shown to be inversely correlated with sensitivity of numerous cancer cell lines to ferroptosis [36]. 

The importance of ferroptosis in the management of neoplastic diseases is well recognized. Cancer cells harbor an increased iron pool and yet manage to escape ferroptosis, due to the fine balance between iron and thiols [37]. However, the fact that neoplastic cells have higher requirements of iron favors ferroptosis as an anticancer process opposing tumor development. Indeed, ferroptosis has been reported as one of the underlying mechanisms in the inhibition of numerous malignancies [38,39]. 

IONPs were found to reduce cellular antioxidant defense systems (e.g., activities of SOD, GSH, and catalase) and to induce ROS generation, lipid peroxidation, and caspase-3 activity promoting breast cancer cell death [40]. Exposure of human hepatocarcinoma cells to IONPs leads to the formation of 8-oxo-2’-deoxyguanosine (8-oxo-dG), and oxidative DNA damage markers. The administration of alternating magnetic field (AMF) induced magnetic hyperthermia and further contributed to an elevated 8-oxo-dG level and malignant destruction [41].

One of the adaptive mechanisms of tumor cells is adaptation to reduced levels of oxygen upregulating the expression of HIF-1. Sanazole, a hypoxic cell radiosensitizer, in a complex with IONP and the cytotoxic drug Berberine reduced the tumor volume of Dalton lymphoma ascites in a murine model [42]. The same study suggested that the observed effect was due to the downregulation of HIF-1 and the upregulation of extrinsic apoptotic pathway. 

Recently, Sang and coworkers developed ferroptosis amplifier nanodevices for cancer treatment. Mitochondria targeted the nanophotosensitizer complex containing superparamagnetic IONPs (SPIONs), and when loaded with Sorafenib, exhibited antitumor activity by inducing ROS formation, lipid peroxidation, and ferroptosis of therapy resistant epithelial-to-mesenchymal transition cells [43]. The same group later described another mitochondrial membrane that targeted a photosensitive nanodevice. Black hole quencher-based fluorescence loaded with magnetic IONPs and Sorafenib was intracellularly disassembled by GSH and anchored to the mitochondrial membrane. Near-infrared (NIR) laser irradiation triggered ferroptosis and inhibited tumor growth both in vitro and in vivo [44]. A variety of iron containing nanopraticles that improve photodynamic therapy (PDT) effects have been successfully synthesized [45,46]. Antitumor activity of IONPs can be enhanced by coating, as in the case of maghemite coated with poly(N,N-dimethylacrylamide) [47]. 

## 4. The Lipid Peroxidation Product 4-Hydroxynonenal

In addition to the above-mentioned importance of iron-induced and ROS-mediated lipid peroxidation and LO● in ferroptosis of neoplastic cells, lipid peroxidation also has an important role in cell regulation. The peroxidation of polyunsaturated fatty acids (PUFAs) and subsequent decomposition of lipid hydroperoxides (LOOH) leads to formation of reactive aldehydes, among which 4-hydroxynonenal (4-HNE) is of particular interest. Namely, due to its bioactive properties, 4-HNE can either directly or indirectly affect macromolecules and modulate cellular processes and function, thus, resembling bioactivities of ROS [48,49]. Known also as a second messenger of free radicals, which interferes with bioactivities of various cytokines, 4-HNE acts in concentration-dependent and cell-type dependent manners [50,51]. Generally speaking, if present at low, physiological concentrations, 4-HNE mostly acts as a growth regulating factor affecting proliferation, differentiation, and antioxidant capacities of the cells, whereas its presence at higher supraphysiological levels usually induces apoptosis or even necrosis. One of major reasons for such multifunctional bioactivities of 4-HNE is its high affinity to bind to proteins, modulating their structure and function and forming a kind of reservoir of 4-HNE-proten adducts, which are hardly metabolized and could offer an explanation for the adverse effects of very different medical remedies, either synthetic or of natural origin [49,52,53,54]. It is commonly believed that 4-HNE possesses mutagenic and carcinogenic affects, and thus resembles negative carcinogenic activities of free radicals. However, due to the altered lipid metabolism and hypoxia, cancer cells develop different antioxidant capacities as compared with their counterpart non-malignant cells, but eventually that makes them more susceptible to the cytotoxic effects of 4-HNE [55,56,57]. Complex interactions between invading cancer and surrounding tissue can lead to the enhanced production of 4-HNE by normal, stromal, and especially inflammatory cells. Then, elevated 4-HNE acts as a kind of anticancer substance [58,59]. The fact that all rapidly proliferating cells, including those that are malignant, require higher levels of iron than is needed for the resting cells, while the iron-triggered and self-catalyzed chain reaction of lipid peroxidation generates 4-HNE, could present novel opportunities to use iron and other transition metals as potential anticancer substances, especially if packed in the form of relatively stable micro- or nanoparticles [60]. 

## 5. Iron-Containing Nanoparticles in Oncotherapy

**Figure 2:**
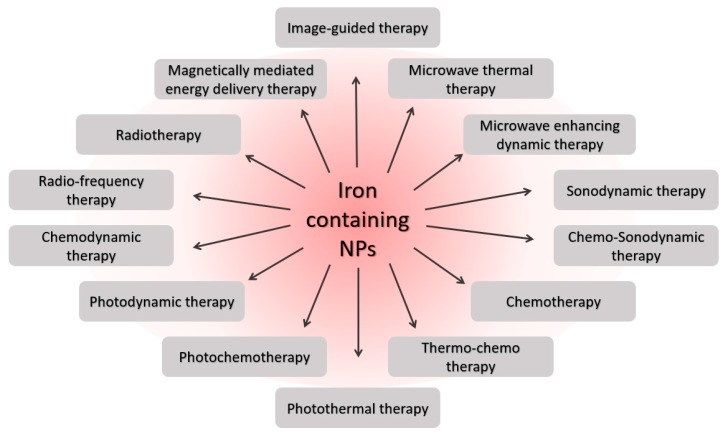
Biomedical therapies involving iron containing nanoparticles.

Ferroptosis, as a promising mechanism for the demise of cancer cells, has become a promising strategy in cancer nanomedicine. Due their advantages, iron-containing nanoparticles are used in numerous nanomedicine strategies in oncology (Figure 2). 

Chemodynamic therapy (CDT) with a high specificity towards a tumor microenvironment (TME) is a promising strategy for the management of cancer. The anticancer properties of CDT are due to their ability to promote conversion of endogenous H_2_O_2_ via Fenton reaction into ●OH triggering lipid peroxidation and ferroptosis of cancer cells [61,62]. In recent years, an effort has been made to develop nanoparticles with encapsulated iron for ferroptosis assisted CDT. Conjugated polymer nanoparticles (CPNPs) containing iron(III) and targeted to endothelin-B receptors selectively trigger ferroptosis of melanoma cells with no significant effect on normal cells [63]. Wang and colleagues developed redox- and light-responsive (RLR) nanoparticles that contained iron and were programmed to degrade and self-activate in an acidic environment with excessive glutathione, as is the case for a tumor microenvironment (TME). The constructed RLR nanoparticles were loaded with doxorubicin (DOX) and modified with iRGD peptide that binds to α_v_β_3_ integrins. Such nanoparticles showed specificity in targeting integrin overexpressing cancer cells as compared with normal cells [64]. Further specificity towards cancer cells has been confirmed in the in vivo tumor model. 

Magnetic hyperthermia is another important application of iron containing nanoparticles for cancer treatment. Magnetic hyperthermia is based on the effects of AMF on the magnetization properties of iron causing a heat release in the surrounding space. The simultaneous action of chemodynamic therapy with magnetic-photo hyperthermia has an inhibitory effect on tumor progression, as reported for Fe_3_O_4_-Pd Janus nanoparticles [65]. The mechanism of anticancer activity included both amplified magnetic-photo heating, as well as elevated ROS production and ferroptosis of cancer cells. Furthermore, nanotheranostic potential of iron oxide nanoparticles was shown for α-Fe_2_O_3_ nanoparticles coated with ultrasmall gold (Au) nanoseeds. The α-Fe_2_O_3_ with Au nanoparticles resembled good photothermal efficacy and upon irradiation, with low laser power, yielded ROS generation and DNA damage, suppressing tumor growth [66]. Compared to ferromagnetics, superparamagnetic iron oxide nanoparticles generate more heat and are extensively used in cancer therapy. Superparamagnetic properties of iron oxide nanoparticles have shown promising results in the hyperthermia treatment of solid tumors such as prostate cancer [67,68] and glioblastoma [69,70]. Impressively, almost complete glioblastoma regression was observed after multiple applications of magnetic hyperthermia [70]. For further reading on the medical applications of SPIONs, a comprehensive review has been published last year [71]. Xiong and colleagues have recently developed a nanosystem containing DOX, FeCl_3_, and tannic acid. Photothermal excitation triggered drug release from the nanosystem, while laser irradiation promoted ROS generation, lipid peroxidation, ferroptosis, reduced intracellular glutathione (GSH) level, and inhibited tumor progression, as shown both in vitro and in vivo on an ER+ breast carcinoma model [72].

The ability of polydopamine nanoparticles to chelate metal ions has been used in developing ultrasmall poly(ethylene glycol) (PEG)-modified polydopamine nanoparticles containing either Fe^2+^ or Fe^3+^ [73]. Such nanoparticles showed potent anticancer activity by ferroptosis via induction of ROS formation and lipid peroxidation and inhibition of GPX4 activity. Furthermore, the specific design of the study identified that Fe^2+^ and Fe^3+^ ion preferred anticancer mechanisms. Nanoparticles containing Fe^2+^ had a preference for ROS induced ferroptosis, whereas those containing Fe^3+^ had a preference for lipid peroxidation-dependent ferroptosis [73].

Recently, nanozymes with iron containing nanoparticles have been demonstrated to be good tools for cancer therapy. Nanozymes are nanomaterials exhibiting intrinsic enzyme-like properties. The peroxidase-like activity of iron containing nanoparticles was first reported in 2007 [74]. To date, iron containing nanozymes with various enzyme mimetic properties have been reported, such as catalase, peroxidase, or SOD-like activities. The classification of nanozymes, together with their catalytic properties and application, have been recently described in an excellent review by Huang and colleagues [75]. One example of nanozymes used for the anticancer strategies, is the recently developed nanozymes biodegradable in the acidic environment with peroxidase-like catalytic activity having microwave enhancing dynamic therapy (MEDT) and microwave thermal therapy (MTT) effects [76]. Microwave irradiation accelerated ●OH generation in the tumor microenvironment. Novel nanozymes, under microwave irradiation, were able to locally increase temperature, as well as promote ROS formation, in particular ●OH, leading to malignant cell destruction and inhibition of a tumor. 

The scientific community is continuously making efforts to improve current, or developing novel more efficient, nanosystems for oncotherapy. Table 1 highlights the recent animal studies with iron nanodevices for cancer therapy by altering cellular redox homeostasis.

## 6. Approaches to Attenuate the Effects of Iron-Containing Nanoparticles on Healthy Cells and Tissue

In addition to the beneficial anticancer activities of iron-containing nanoparticles, one should also consider the potential adverse effects for healthy tissue. Toxicological potential of a variety nanoparticles [87], including the ones containing iron, have been reported [88]. Most of the metal nanoparticles induce generation of ROS and affect cellular redox mechanisms that could lead to their genotoxicity [89]. Iron-containing nanoparticles were shown to induce oxidative stress and dermal toxicity in vitro [88]. Janus Fe_3_O_4_-TiO_4_ nanoparticles, a potential agent for magnetic resonance imaging and PDT, when used at a higher dose (25 μg/cm^2^), alter cellular redox homeostasis of normal hepatic cells inducing lipid peroxidation while decreasing cell viability and ATP levels. However, if used at a lower dose (6.25 μg/cm^2^), such effects are not observed [90]. Additionally, particle size and different coatings on their surface can be detrimental for their activity [91]. Therefore, it is of crucial importance to develop strategies that will attenuate the adverse effects of iron containing nanoparticles for healthy tissue. Moreover, one should bear in mind that cytotoxicity of nanomaterials is dose dependent and concentrations that showed beneficial effects in vitro could have no effect or adverse effects in vivo and could also differ depending on the target tissue. Supplementation with antioxidants, such as thymoquinone, showed promising effects both in vitro and in vivo [92]. Indocyanine green used for PDT was found to be more stable and safer for healthy tissue when loaded to 3-aminopropyltrimethoxysilane coated cationic SPIONs and used as PTT and PDT dual therapy [77]. Functionalization of the IONPs surface with small molecules or polymers could improve IONPs stability and biocompatibility [1,93]. Functionalization can also improve or add new properties to IONPs such as antioxidant and antimicrobial activity [94], enabling thermal therapy [93], or promoting ROS-induced anticancer activity [95].

## 7. Conclusions

Iron is necessary for numerous physiological processes but could also be an important trigger of oxidative stress, especially of lipid peroxidation, thus, contributing either to carcinogenesis or to toxicity of anticancer treatments. Hence, ferroptosis acts as a potential natural anticancer defense mechanism that uses iron to generate ROS and lipid peroxidation and eventually destroy cancer cells. Furthermore, recent findings indicate that the end products of lipid peroxidation, notably 4-HNE, could be generated by non-malignant cells as a defense mechanism against cancer complementary to ferroptosis. Although numerous promising iron-containing nanoparticles were developed for experimental radio-, chemo-, and photodynamic, as well as magnetic dynamic nanosystems that induce oxidative stress and ferroptosis, they do not exploit selective cytotoxicity of 4-HNE as a potentially novel anticancer activity principle. Namely, binding of 4-HNE to (extra)cellular proteins could result in persistent lipid peroxidation selectively destroying cancer cells due to the differences in lipid metabolism and antioxidant systems between cancer and non-malignant cells. Therefore, nanosystems should be developed that use iron to trigger ferroptosis and target lipid peroxidation as anticancer options of integrative biomedicine utilizing the benefits of advanced nanotechnologies and of natural anticancer defense mechanisms of the host.

## Figures and Tables

**Table 1:** Recent animal studies with variety of iron containing nanoparticles for cancer therapy by altering cellular redox homeostasis.

Tumor Type	Animal Tumor Model	Type of Nanoparticles	Reported Therapy	Effect	Ref.
Breast cancer	Breast tumor xenograft mouse model	RLR nanoparticle composed of Fe_3_O_4_ nanoparticles and a nanoflower-like MnO_2_ shell	Chemodynamic therapy	Nanoparticles are degraded under acidic environment and excessive GSH yielding accelerated ROS production and tumoricidal effect.	[64]
Breast cancer	4T1 tumor-bearing mice	Fe_3_O_4_-Pd Janus nanoparticles (JNPs)	Simultaneous magnetic-photo hyperthermia and chemodynamic therapy	Dual exposure of nanoparticles to AMF and laser irradiation induces enhanced temperature and ROS generation that are via Fenton reaction converted to OH●.	[77]
Breast cancer	4T1 tumor-bearing mice	α-Fe_2_O_3_ nanoparticles coated with ultrasmall gold nanoseeds	Magnetic resonance imaging, photothermal therapy and radiosensitization	Upon NIR irradiation nanoparticles showed enhanced photothermal therapy and sensitized radiotherapy by inducing ROS formation and tumor inhibition.	[66]
Breast cancer	4T1 tumor-bearing mice	Ultrasmall PEG-modified polydopamine nanoparticles containing Fe^2+^/^3+^	Chemotherapy, Ferroptosis therapy	Fe^2+^ containing nanoparticles induce ROS dependent ferroptosis while Fe^2+^ containing nanoparticles induce lipid peroxidation-dependent ferroptosis.	[73]
Breast cancer	4T1 tumor-bearing mice	Nanoparticles porphyrin-based metal–organic framework and MnFe_2_O_4_ nanoparticles as the nanoenzyme	Enhanced Photodynamic Therapy	Nanodevice exhibits catalase-like and GPX-like activity, In the tumor, upon irradiation, continuously promotes ROS formation via Fenton reaction, and reduces GSH modulating tumor microenvironment.	[78]
Breast cancer	4T1 tumor-bearing mice	Silica nanoparticles with MnO_2_ nanoparticles and FeCO	Synergistic Gas therapy (GT) and chemodynamic therapy (CDT)	Under acidic environment MnO_2_ promotes ROS that further triggers decomposition of FeCO into CO.	[79]
Breast cancer	MCF-7 tumor-bearing mice	Nanogel loaded with magnetic IONPs and 10-hydroxy camptothecin	Enhanced photothermal-chemotherapy	Nanogel represents a good anticancer drug delivery system and can also serve as nanocarrier for photothermal therapy due to its absorption at NIR region. Furthermore, magnetic IONPs in the presence of macrophages promote ROS formation.	[80]
Breast cancer	4T1 tumor-bearing mice	Nanosystem containing Fe(OH)_3_ modified upconversion nanoparticles	Synergetic chemo- and photodynamic therapy	INR irradiation promotes ROS formation in cancer cells.	[81]
Breast cancer	4T1 tumor-bearing mice	IONPs modified with glucose oxidase and polydopamine	Photothermal therapy	NIR irradiation induces heat generation and formation of H_2_O_2_. H_2_O_2_ is then via Fenton reaction converted to OH● inducing apoptosis of cancer cells.	[82]
Breast cancer	4T1 tumor-bearing mice	ROS nanoreactor based on core-shell-structured iron carbide nanoparticles	Magnetic Resonance Imaging Guided Cancer Therapy	In the acidic tumor microenvironment Fe^2+^ are released in acidic environments where, via Fenton reaction, generate OH● and inhibit tumor.	[83]
Breast cancer	4T1 tumor-bearing mice	FeOOH nanoparticles coated with poly(norepinephrine) and loaded with artemisinin (Art)	Photothermal-chemical combination therapy	Exposure to NIR promotes heat generation and synchronous release of iron ions and Art in the acidic tumor microenvironment promotes generation of ROS and subsequent generation of OH● via Fenton reaction having high toxicity for tumor and low for normal tissue.	[84]
Breast cancer	4T1 tumor-bearing mice	Mitochondrial membrane targeted nanophotosensitizer complex containing SPION and sorafenib	Ferroptosis as cancer therapy	Activated nanoparticles consume GSH, induce excessive ROS production and release SPION and sorafenib, promoting ferroptosis. Efficacy was also shown for the drug resistant in vitro model.	[43]
Breast cancer	4T1 tumor-bearing mice	Mitochondrial membrane targeted nanophotosensitizer complex containing magnetic IONPs and sorafenib	Ferroptosis as cancer therapy	Activated nanoparticles downregulate GPX-4 and xCT inducing ferroptosis.	[44]
ER+ breast cancer	MCF7 tumor xenograft model Balbc	Drug-organics-inorganics self-assembled (DFTA) nanosystem with DOX, FeCl_3_ and tannic acid	Chemotherapy, Photothermal therapy and Ferroptosis therapy	Photothermal excitation triggers DOX release, activates SOD-like reaction and reduces GSH through excessive ROS production.	[72]
Glioma	Ectopic glioma tumor-bearing mice	Fe_3_O_4_-IR806 superparticles	Photothermal-photodynamic therapy	Photothermal conversion efficacy upon NIR irradiation was enhanced and promoted excessive ROS formation exhibiting tumor toxicity.	[85]
Hepato-cellular carcinoma	H22-tumor bearing mice and HepG2 tumor-bearing nude mice	Nanozymes containing Fe-metal organic framework nanoparticles	Microwave enhancing dynamic therapy, Microwave thermal therapy	Microwave irradiation promotes excessive ROS formation, in particular OH●, inducing cell death. In the presence of gold nanoclusters, the same can have application in imaging and microwave thermal therapy.	[76]
Hepato-cellular carcinoma	H22-tumor bearing mice and HepG2 tumor-bearing nude mice	PEG-modified nanoparticles loaded with photosensitizer and MnFe_2_O_4_ and silica upconversion nanoparticles	Photodynamic therapy	Loading efficiency of photosensitizer is increased NIR irradiation activates luminescence form upconversion nanoparticles yielding activation of photosensitizer and consequent ROS formation that take part in Fenton reaction eliciting tumoricidal effect.	[46]
Lung adenocarcima	A549 tumor-bearing nude mice	Modified IONPs with β-lapachone encapsulated in the nanostructure formed by H_2_O_2_-responsive polyprodrug and pH-responsive polymer (LaCIONPs)	Chemo/chemodynamic combination therapy	Acidic environment of tumor cells triggers LaCIONPs decomposition triggering excessive H_2_O_2_. H_2_O_2_ further via Fenton reaction produces OH● and also activates the release of drug eliciting tumoricidal effect.	[86]
Prostatic cancer	PC3 tumor-bearing nude mice	γ-Fe_2_O_3_ with copper sulfide shell	Photothermal Therapy, Magnetic Hyperthermia and Photodynamic Therapy	NIR exposure and magnetic stimulation promotes heat generation and ROS formation with tumoricidal effects.	[68]
